# Assessment of bone marrow edema on dual-energy CT scans in people with diabetes mellitus and suspected Charcot neuro-osteoarthropathy

**DOI:** 10.1007/s00256-024-04714-3

**Published:** 2024-06-04

**Authors:** Carlijn M. B. Bouman, Marieke A. Mens, Ruud H. H. Wellenberg, Geert J. Streekstra, Sicco A. Bus, Tessa E. Busch-Westbroek, Max Nieuwdorp, Mario Maas

**Affiliations:** 1 Amsterdam UMC, Radiology and Nuclear Medicine, Location University of Amsterdam, Meibergdreef 9, Amsterdam, The Netherlands; 2 Amsterdam UMC, Biomedical Engineering and Physics, Location University of Amsterdam, Meibergdreef 9, Amsterdam, The Netherlands; 3 Amsterdam UMC, Location University of Amsterdam, Rehabilitation Medicine, Meibergdreef 9, Amsterdam, The Netherlands; 4 Amsterdam UMC, Internal and Vascular Medicine, Location University of Amsterdam, Meibergdreef 9, Amsterdam, The Netherlands; 5 Amsterdam Movement Sciences, Rehabilitation and Development, Amsterdam, The Netherlands

**Keywords:** Dual-energy CT, Bone marrow edema, Charcot foot, Diabetic foot, Computed Tomography

## Abstract

**Objective:**

This study aimed to quantitatively assess the diagnostic value of bone marrow edema (BME) detection on virtual non-calcium (VNCa) images calculated from dual-energy CT (DECT) in people with diabetes mellitus and suspected Charcot neuro-osteoarthropathy (CN).

**Materials and Methods:**

People with diabetes mellitus and suspected CN who underwent DECT of the feet (80kVp/Sn150kVp) were included retrospectively. Two blinded observers independently measured CT values on VNCa images using circular regions of interest in five locations in the midfoot (cuneiforms, cuboid and navicular) and the calcaneus of the contralateral or (if one foot was available) the ipsilateral foot. Two clinical groups were formed, one with active CN and one without active CN (no-CN), based on the clinical diagnosis.

**Results:**

Thirty-two people with diabetes mellitus and suspected CN were included. Eleven had clinically active CN. The mean CT value in the midfoot was significantly higher in the CN group (-55.6 ± 18.7 HU) compared to the no-CN group (-94.4 ± 23.5 HU; *p* < 0.001). In the CN group, the difference in CT value between the midfoot and calcaneus was statistically significant (*p* = 0.003); this was not the case in the no-CN group (*p* = 0.357). The overall observer agreement was good for the midfoot (ICC = 0.804) and moderate for the calcaneus (ICC = 0.712). Sensitivity was 100.0% and specificity was 71.4% using a cutoff value of -87.6 HU.

**Conclusion:**

The detection of BME on VNCa images has a potential value in people with diabetes mellitus and suspected active CN.

## Introduction

Charcot neuro-osteoarthropathy (CN) is one of the most severe complications of diabetic peripheral polyneuropathy. CN is relatively uncommon with an incidence of 0.1–0.3% in the diabetic population and is therefore often not a diagnosis that physicians consider at first [1, 2]. This condition can be described as a non-infectious inflammatory process, primarily in the midfoot leading to a collapse of the foot arch and, eventually, a rocker bottom foot, increasing the risk of foot ulceration [3–5].

To prevent major foot deformity, rapid immobilization and offloading are warranted [6, 7]. However, the diagnosis of CN is often delayed, as the disease is frequently not recognized in an early stage [7–9]. An active CN foot is defined as a red, warm and swollen foot with osseous abnormalities on imaging [10]. A plain radiograph is performed as the initial form of imaging and classifications systems such as the Brodsky and Eichenholtz classification are based on plain radiographs [10–13]. One of the key findings indicating inflammation due to active CN is bone marrow edema (BME) [14, 15]. The location and distribution of BME can aid in differentiating CN from other diseases such as osteomyelitis [12]. However, radiographs cannot visualize bone marrow [11]. An imaging modality that can detect BME is magnetic resonance imaging (MRI). However, MRI often has long waiting times, not beneficial in patients with acute conditions, and has downsides such as high costs, contra-indications in people with certain implants or claustrophobia and might be uncomfortable for people with severe polyneuropathy who have difficulty laying still for extended periods of time. Since there is a need for an early and accurate diagnosis, virtual non-calcium (VNCa) images calculated from dual-energy Computed Tomography (DECT) could be of value for rapid detection of bone marrow edema in the midfoot related to CN. DECT has shown to have a high sensitivity and specificity for detecting BME in multiple pathologies, including occult fractures, osteomyelitis and forms of arthritis [16–21].

This study aimed to investigate if DECT with VNCa can detect BME in the midfoot in people with diabetes mellitus and suspected active CN.

## Materials and Methods

### Study population

Informed consent was waived by the Medical Ethics Review Committee of the Amsterdam UMC. Inclusion criteria were a diagnosis of diabetes mellitus type 1 or 2, clinically suspected active CN and diabetic peripheral neuropathy. Individuals were classified as having clinically suspected active CN if they presented with an edematous and erythematous foot and an elevated foot temperature of > 2 degrees Celsius compared to the unaffected foot [22]. Exclusion criteria were concomitant foot diseases (e.g. osteomyelitis), recent trauma and unavailable digital patient files. Two clinical study groups were formed: active CN (CN group) and no active CN (no-CN group) depending on the final clinical diagnosis of a multidisciplinary team of medical specialists. This diagnosis was based on clinical data, presence of osseous abnormalities (e.g. fractures, joint subluxation or dislocation) on plain radiographs or DECT (the presence of BME on VNCa was not used in this diagnosis) assessed by a musculoskeletal radiologist, the need for a total contact cast, and the course of the disease during follow-up with a minimum of 3 months. All relevant diagnostics were performed on the same day that a patient presented with clinical signs of active CN. We adhered to the recommendations for diagnosis that are listed in the IWGDF guideline regarding CN [10]. MRI was not available, due to long waiting times in clinical practice. All DECT scans of the feet performed between January 2018 and September 2022 of people being treated at the Amsterdam UMC were retrospectively screened. A sub-analysis was performed in people without a previous diagnosis of CN, thus CN de novo. People with a history of CN might have fragmentation of the midfoot which might influence the results. It is therefore important to know if there is a significant difference CT value in this sub-group as well.

### DECT protocol

A dual-source CT scanner (SOMATOM Force; Siemens Healthcare, Forchheim, Germany) was used (80kVp (tube A) and Sn150kVp (tube B)). No contrast agents were used. CT parameters included: collimation of 0.6 mm, rotation time of 0.5 s, slice thickness and increment of 1.5 mm, mean dose length product (DLP) of 164.1 mGy*cm (range 113.7—246.0 mGy*cm), mean CT dose index volume (CTDIvol) of 6.2 mGy (range 3.9—8.5 mGy), and a medium smooth kernel (Qr54d or Qr40d). Automatic attenuation-based tube current modulation was applied on both tubes (150 reference mAs (tube A) and 380 reference mAs (tube B)).

### DECT image post-processing

Three image data sets were reconstructed from each DECT scan: a weighted average data set, a 80kVp dataset and a Sn150kVp dataset. Subsequently, all scans were processed using a three-material decomposition algorithm, differentiating between yellow bone marrow, red bone marrow, and bone mineral (SyngoVia VB40; Siemens Healthcare, Forchheim, Germany), a lower threshold of 50 Hounsfield unitis (HU) and a maximum of 800 HU. This allowed for the creation of VNCa-images by subtracting calcium. Densities between -150 HU and 100 HU were used on the color-coded bone marrow maps. BME is around 0 HU and appears as green on the color-coded map. On the color-coded map, higher values appear yellow turning eventually red and lower values appear blue gradually turning purple.

### Quantitative measurements

All scans of the feet were independently assessed by two medically trained researchers (C.M.B. and M.A.M.). The researchers were instructed and supervised to conduct the measurements by a musculoskeletal radiologist (M.M.) with over 30 years of experience in diabetic foot disease. The researchers were blinded for the radiology report and clinical information.

The anatomical locations where CN is most prevalent can be described according to the classification of Brodsky-Trepman [23, 24]. This is a reliable classification and important to the clinical communication about CN [13]. The Brodsky classification describes five types of CN according to their anatomical location. Type 1 (tarsometatarsal and naviculocuneiform joints) and type 2 (subtalar, talonavicular or calcaneocuboid joints) account for around 90% of all CN cases [25]. We decided to limit the measurement locations to five bones in the midfoot, in accordance with Brodsky type 1 and 2. This makes the measurements representative for a clinical setting. CN of the hindfoot, Brodsky-Trepman type 3, accounts approximately for 10% of the cases, mostly in the ankle (type 3A). CN of the calcaneus (type 3B) can lead to an avulsion fracture of the tendon-Achilles [23, 25]. The calcaneal body can be used as a reference standard due to its size and the rarity of CN presenting in the calcaneus or affecting the measurements in the calcaneus body.

**Fig. 1:**
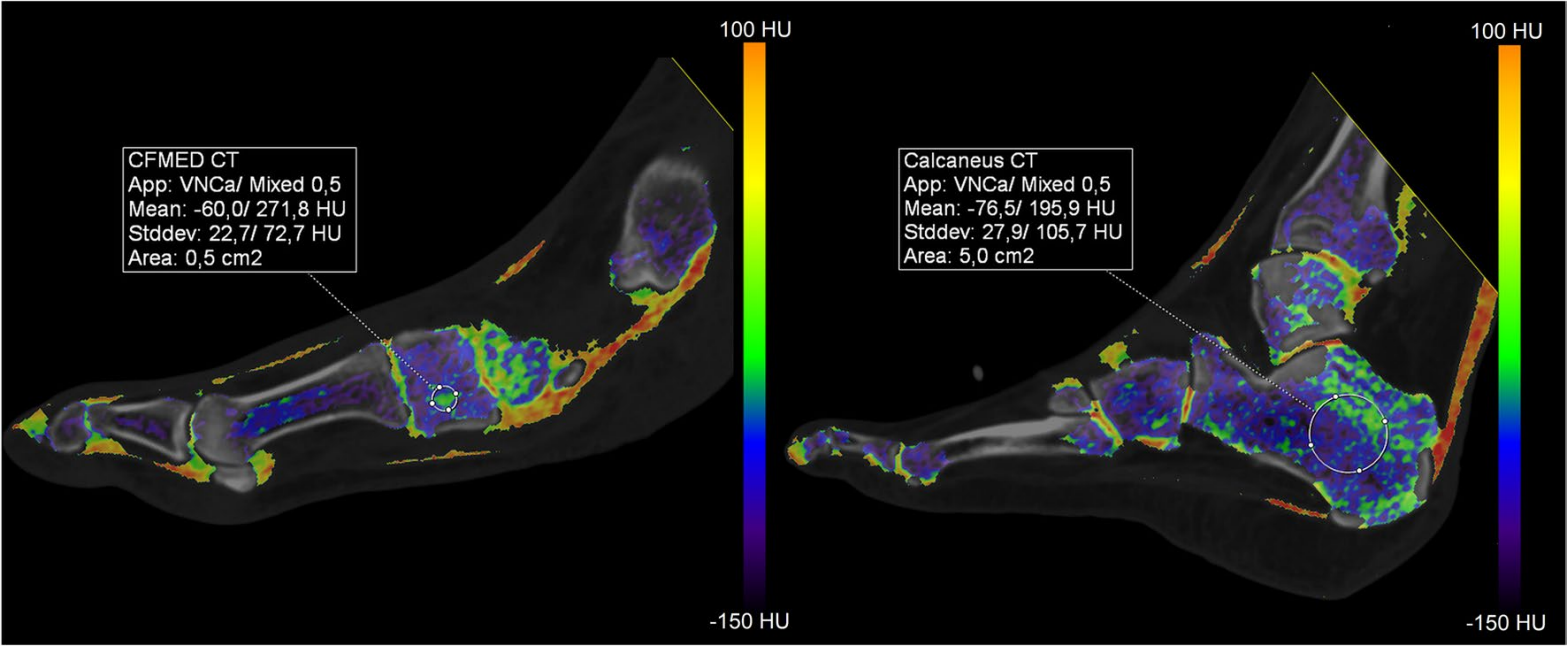
ROI placement in the medial cuneiform bone (left) and in the calcaneus (right) on the VNCa map

A circular region of interest (ROI) of 0.5 cm^2^ was manually placed in the five locations in the midfoot: medial, intermediate and lateral cuneiform bones, cuboid bone and navicular bone (Fig. 1). The exact placement of the ROI was not fixed, but based on the color-coded map. The ROI was placed in a region where the color-coded map showed a lot of green (indicative of elevated CT values). When the color-coded map showed no green areas in one of the bones, the region that was estimated to contain the highest CT value was used. This was the region that contained the least amount of purple color-coded areas (indicative of very low CT values). ROIs were placed in the sagittal reconstruction and with a minimum of 2 mm from the cortex. The contralateral calcaneus (or, if unavailable, the ipsilateral calcaneus) was used as a reference location. A large ROI of 5.0 cm^2^ was placed in the middle of the calcaneus (Fig. 1), thereby providing a better representation of the average CT values. If a bone was fragmented and a circular ROI could not be placed, a freehand ROI was drawn. The mean CT value inside the ROI was noted. Afterwards, the measurements of both observers were pooled and the averaged CT value of the five measurements was used for further calculations. To evaluate if there are differences between the measurements in the five midfoot bones and to assess if one bone is more suitable for these kinds of measurements, an analysis between the two study groups with the separate measurements in the different bones was performed as well.

### Qualitative measurements

**Fig. 2:**
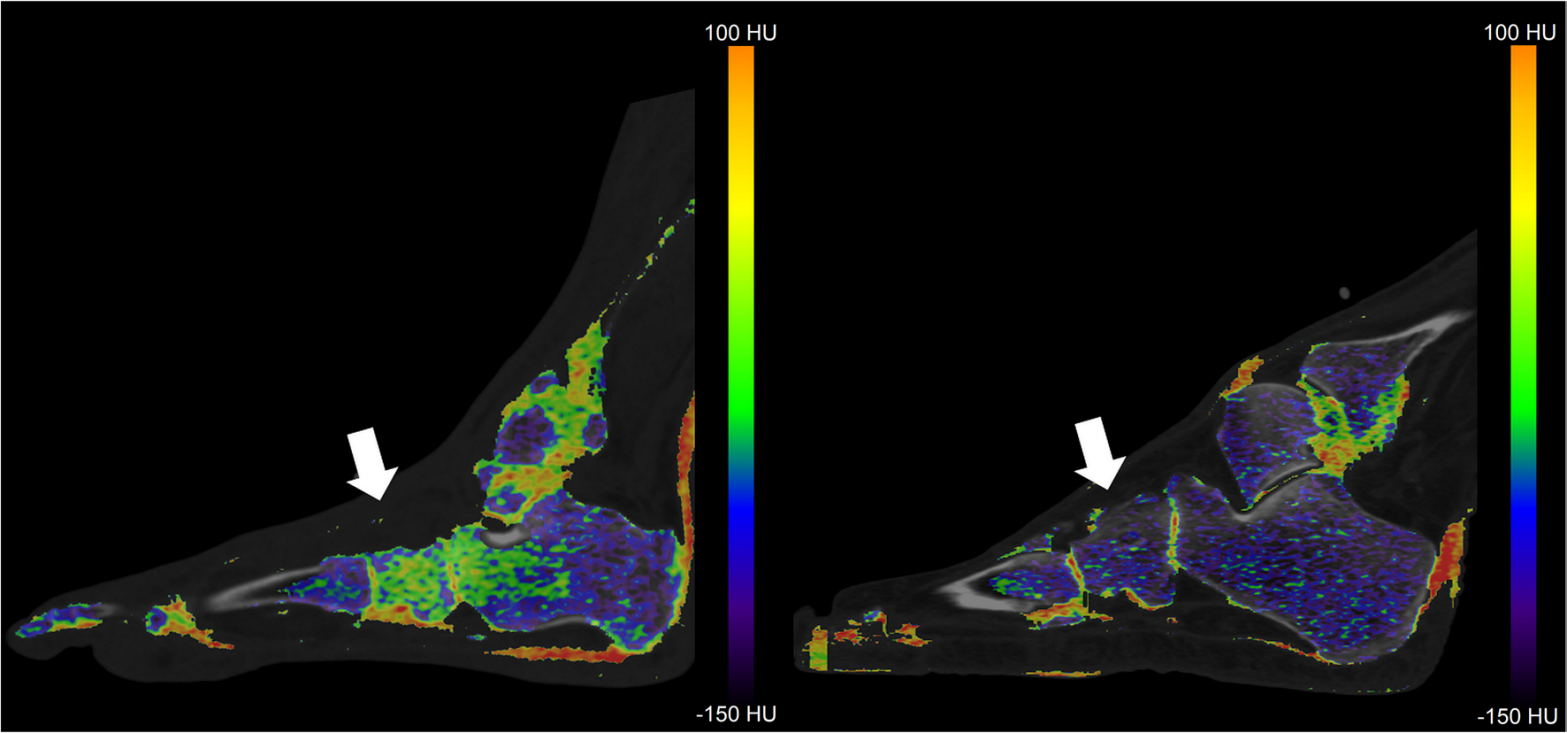
Sagittal VNCa images of a foot with active CN (left) and without active CN (right). The white arrows point to the cuboid bone. In the foot with active CN the average CT value measured in the cuboid bone higher, shown as green or yellow indicating the presence of BME

The VNCa images were assessed qualitatively by a physician with three years of research experience in musculoskeletal radiology (M.A.M.) and an experienced musculoskeletal radiologist (M.M.) for the presence of BME indicative of active CN. The readers were blinded for the radiology report and clinical information and used the color-coded map for the assessment (Fig. 2). The presence of BME was considered positive if one or both reviewers considered BME to be present. Inter observer agreement was assessed.

### Statistical analysis

SPSS (version 26.0, IBM, Armonk, New York, USA) was used for the statistical tests. The Kolmogorov–Smirnov test was used to test for normality. A student’s t-test or Mann–Whitney U test was used to test for significance between the groups and between the average CT values in the midfoot and reference location. The midfoot bones were also analyzed separately between the groups, as well as the highest measured CT value regardless of location. The Chi-squared test was conducted to test the significance for binary data. A *p-*value < 0.05 was considered as statistically significant. The intraclass correlation coefficient (ICC) or Cohen’s kappa were used to determine inter-observer agreement for respectively the quantitative and qualitative measurements. An ICC < 0.5 was considered “poor”, 0.5–0.74 “moderate”, 0.75–0.9 “good” and > 0.9 “excellent” [26]. A kappa of < 0 was “poor”, 0–0.20 “slight”, 0.21–0.40 “fair”, 0.41–0.60 “moderate”, 0.61–0.80 “substantial” and > 0.81 “almost perfect” [27]. In addition, a Bland–Altman analysis was conducted. A Receiver Operating Characteristic (ROC) analysis was performed. A cutoff CT value for diagnosing BME was determined using the Youden’s index based on the ROC analysis. Additionally, we calculated sensitivity, specificity, positive predictive value (PPV) and negative predictive value (NPV).

## Results

### Patient population

DECT-scans of 32 people with diabetic peripheral neuropathy and clinically suspected active CN were included (Table 1). In 11 cases, active CN was diagnosed (8 men; 3 women; age 59.8 years ± 13.8 years; BMI 28.7 ± 7.2). In 21 cases, active CN was not diagnosed (12 men; 9 women; age 63.2 years ± 10.2 years; BMI 30.7 ± 5.6). A majority of the people in both groups had a history of type 2 diabetes mellitus (CN n = 9 (82%); no-CN n = 17 (81%)). Two people in the CN group received dialysis and one had a history of osteoporosis. In the no-CN group, one received dialysis and three had a history of osteoporosis. Significantly more people had a history of CN in the no-CN group (CN n = 3 (27%); no-CN n = 15 (71%); p = 0.02) and the time since the diagnosis of diabetes was significantly longer in the no-CN group (CN 14.7 years ± 12.6 years; no-CN 22.0 years ± 10.9 years; p = 0.04). There was fracturing of the midfoot in some people with a history of CN. As a result, it was not always possible to perform the measurements on all bones in the study population.

**Table 1:** Patient demographics

	CN	No-CN	P-value
DECT-scans	11 (34)	21 (66)	-
Sex (male)	8 (73)	12 (57)	0.48
Age (years)	59.8 ± 13.8	63.2 ± 10.2	0.82
BMI (kg/m^2^)	28.7 ± 7.2	30.7 ± 5.6	0.44
Type of diabetes (type 2)	9 (82)	17 (81)	0.90
Dialysis	2 (18)	1 (5)	0.22
Osteoporosis	1 (9)	3 (14)	0.67
HbA1c (mmol/mol)	52 ± 10	62 ± 18	0.10
eGFR (ml/min/1.73 m^2^)	55 ± 32	66 ± 25	0.33
Time since diabetes diagnosis (years)	14.7 ± 12.6	22.0 ± 10.9	0.04*
History of Charcot	3 (27)	15 (71)	0.02*

Data are mean ± SD or n (%); * statistically significant; BMI = body mass index, HbA1c = Hemoglobine A1c, eGFR = estimated glomerular filtration rate; HbA1c and eGFR are taken within 3 months before or after the DECT scan

### Quantitative analysis

**Fig. 3:**
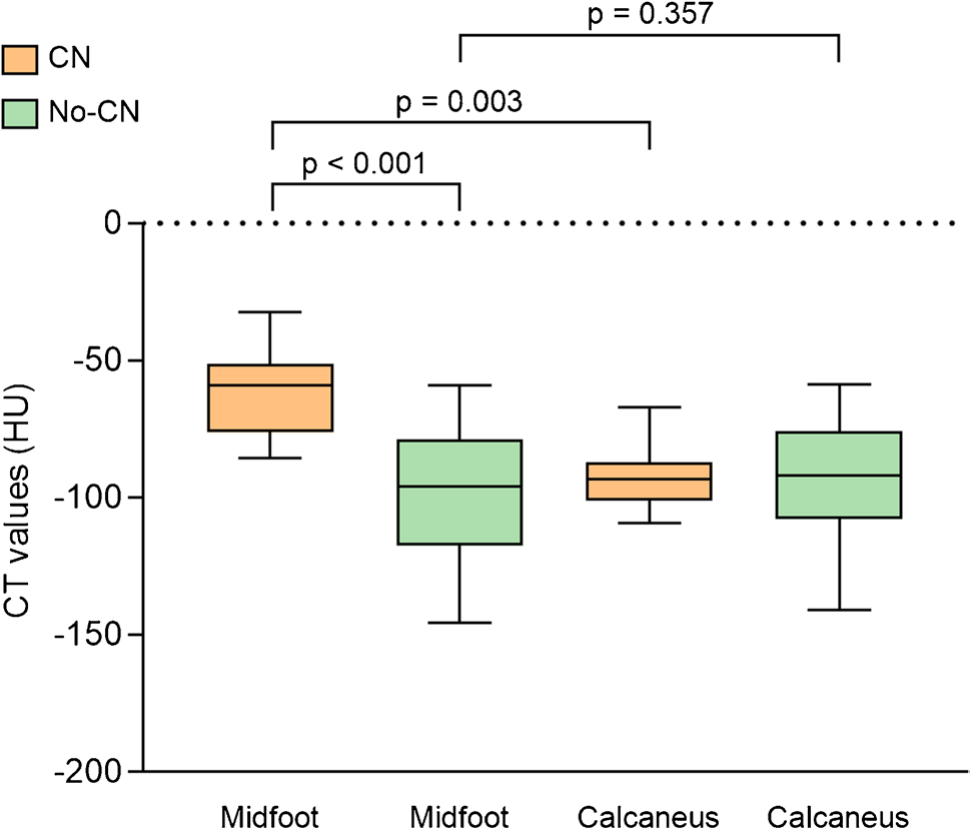
Box-plot of the CT values of regions of interest in the midfoot and calcaneus in both groups

**Fig. 4:**
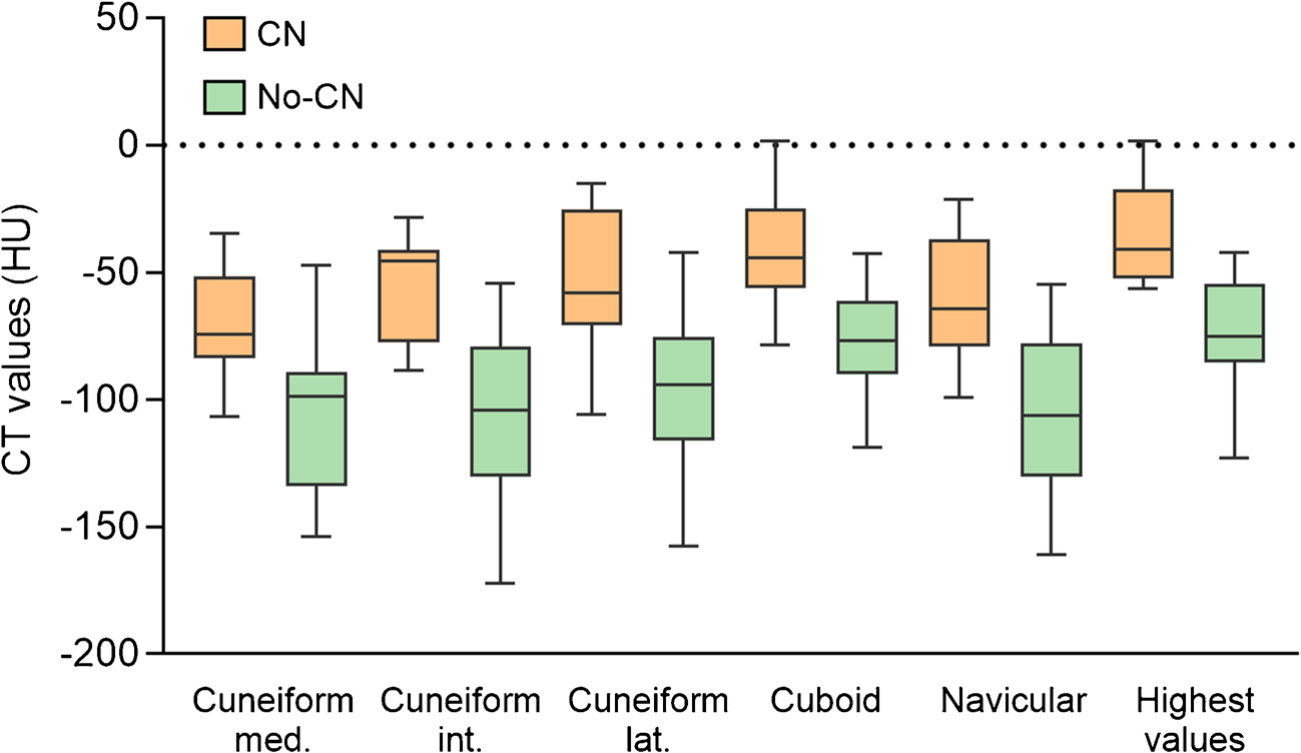
Box-plot of the CT values of the CN and no-CN groups differentiated between midfoot bones. The two rightmost boxes represent the highest values found per patient. There was a significant difference (*p* < 0.001) in all bones between the two groups, including when the highest value was chosen

There was a statistically significant difference in average CT values of the bone marrow in the midfoot between the CN and no-CN groups (-55.6 ± 18.7 HU and -94.4 ± 23.5 HU, *p* < 0.001; Fig. 3). The CT values of the reference location were not significantly different between the CN and no-CN groups (-93.0 ± 11.6 HU and -92.3 ± 20.8 HU, *p* = 0.781). Regarding the CN group, there was a significant difference in CT values between the midfoot and reference location (*p* = 0.003). In the no-CN group, the midfoot showed similar CT values to the reference location (*p* = 0.357). In a sub-analysis of only people without a history of CN, thus suspected for CN de novo, a significant difference between CT values in the midfoot between groups was found (-50.8 ± 19.4 and -114.6 ± 23.3, *p* = 0.003). When the different bones were analyzed individually, a significant difference in CT values in all bones was found between the groups (*p* < 0.001; Fig. 4). The cuboid bone has the smallest range in measured CT values. This could be explained by the large size of the bone compared to the other midfoot bones. This makes it easier to identify a region suitable for ROI placement. The cuboid bone also shows higher CT values than the other bones and was often the bone with the highest CT value. When conducting an ROC analysis on the CT values of the cuboid bone alone, the area under the curve is smaller than when the average of the CT values is used (0.861 vs. 0.935). The Youden’s index shows that -62 HU would generate the optimal cutoff value for the cuboid bone, with a sensitivity of 82% and a specificity of 76%. This means that a cuboid bone with a CT value above -62 HU might be considered a “bone at risk” for fracturing and fragmentation caused by CN.

**Fig. 5:**
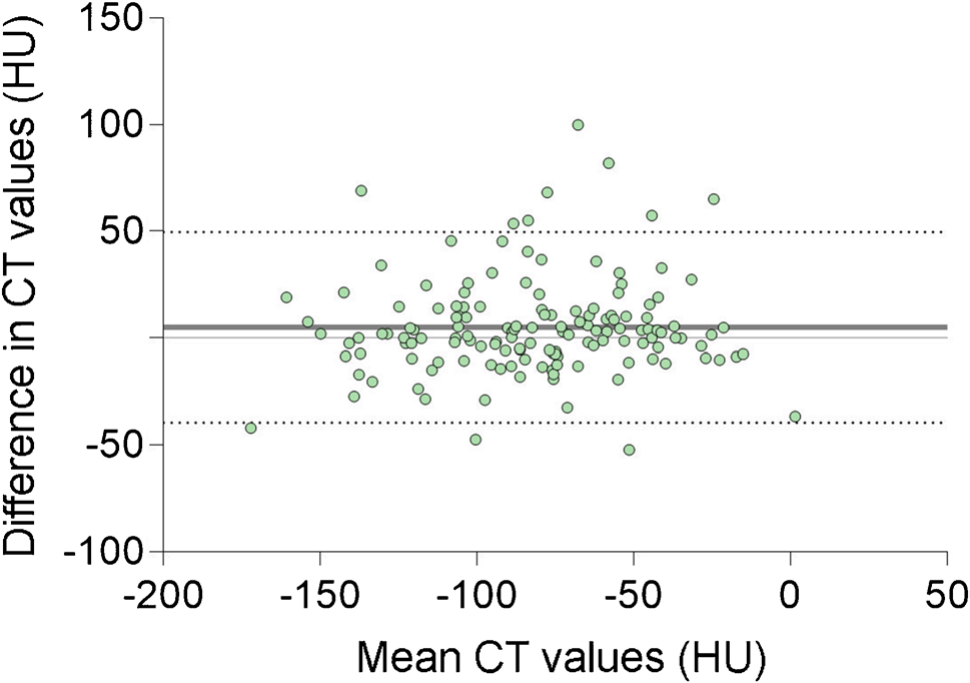
Bland–Altman plot representing the agreement between observers

**Fig. 6:**
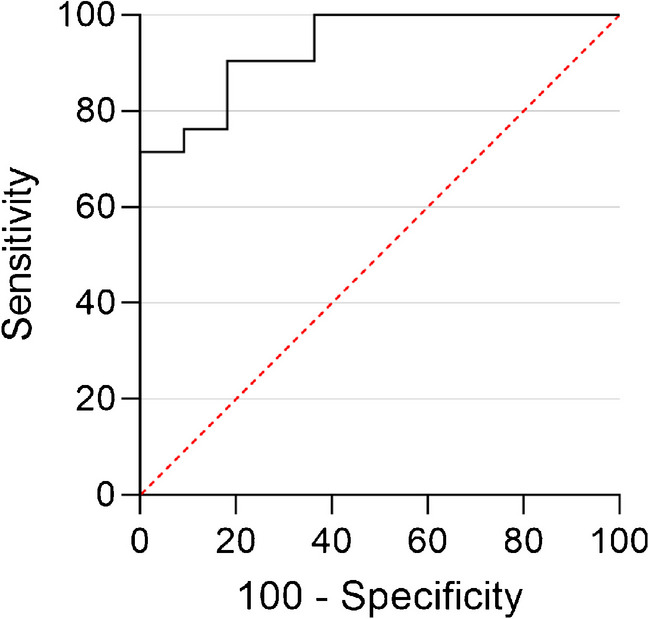
Receiver operating characteristic curves (ROC) of the CT values. The area under the curve was 0.935

Measurements in the midfoot showed a “good” inter-observer agreement (ICC = 0.804) and reference measurements showed a “moderate” inter-observer agreement (ICC = 0.712). A Bland–Altman plot was made to represent the difference between observers in the measurements in the midfoot (Fig. 5). The Bland–Altman analysis showed a bias of 5.1 HU with an SD of 22.7 HU and 95% agreement limits of -39.5 HU and 49.6 HU. Regarding the ROC analysis, the area under the curve was 0.935 (Fig. 6). Sensitivity was 100.0%, specificity was 71.4%, PPV was 64.7% and NPV was 100.0%, using a cutoff value of -87.6 HU.

### Qualitative analysis

BME indicative of active CN was considered as present in 15 of the 32 cases (46.9%). CN and no-CN groups were significantly different regarding BME presence (*p* = 0.004). The Cohen’s kappa was “almost perfect” (κ = 0.81) due to disagreement in 3 cases. Sensitivity was 81.8%, specificity was 71.4%, PPV was 60.0% and NPV was 88.2%.

## Discussion

This study aimed to assess if VNCa images calculated from DECT could be used in the diagnosis of active CN. There were significantly higher CT values in the midfoot of people with active CN when compared to the midfoot of people without active CN. Additionally, the midfoot of people with active CN showed significantly higher CT values than a reference location, which was not the case in people without active CN. In both a quantitative and a qualitative assessment, VNCa images showed a high sensitivity and moderate specificity for detecting BME in active CN.

In this study, a cutoff value of -87.6 HU was found to yield the highest sensitivity and specificity. A condition that is often seen in diabetic foot disease and should be considered with the presence of an erythematous and swollen foot is osteomyelitis related to diabetic foot ulceration. For this condition, a study proposed a cutoff value of -40.1 HU, a much higher cutoff value than we proposed in this study [21]. CN is associated with the development of stress fractures [28]. In a study analyzing DECT in compression fracture of the vertebrae, a cutoff value of -50 HU was used [29]. This cutoff value is also higher than the one we found for CN. The method we used to calculate the CT values of the midfoot could be the reason. We used the average of five measurements in the midfoot. BME was usually not present in all bones of the midfoot when there was a diagnosis of active CN.

An interesting difference in our patient population was that there were significantly more people with a history of CN in the no-CN group. The most likely explanation for this is that the treating physician is more likely to request a DECT scan when a patient was already diagnosed with CN before because they have already proven to be susceptible for this disease. In our sub-analysis we only assessed people with CN de novo. In these cases, the anatomy of the foot has not yet been altered due to previous episodes of disease. This makes the measurements more reliable, because there is no fragmentation in the midfoot. Since fragmentation might influence the results, it is important to know if there is also a significant difference when there was no interference of fragmentation.

A strength of this study is that DECT was evaluated in both a quantitative and a qualitative manner. This provides a broad overview of the application of DECT in people with suspected active CN. Additionally, DECT had not yet been evaluated in people with suspected active CN. The main limitation is the absence of MRI as part of the reference standard. Another limitation is that obtaining quantitative measurements is challenging due to the small size of the bones. If fragmentation occurs, this becomes even more complicated, as the surface area of the fragments is sometimes insufficient to obtain a measurement. In addition, it is more difficult to differentiate bone marrow from the cortex, particularly in the intermediate cuneiform bone. Also, it is possible not all foot bones in a healthy individual have the same CT values. Guggenberger et al. [30] found a gradual increase in CT values from proximal tot distal in the ankle and different cut of values in three areas of the talus. Therefore, it is likely that not all bones in the foot have the same cut-off value. This difference in CT values between bones is also visible in Fig. 4. However, the differences between the bones were not statistically significant. Additionally, there is a small chance that the researchers performing the measurements were biased by certain features on CT, besides BME, that can point to the presence of active CN. This might influence the blinding of the researchers. However, the most apparent features of CN such as fractures or fragmentation of bones can also indicate a history of CN and does not necessarily mean that active CN is present. Changes in density that can indicate active CN are not clearly visible while using the color-overlay. Thus, the chance that this influenced the results is minute. Our final limitation is the relatively small sample size in this study. Even though, diabetic neuropathy is very prevalent in diabetes, CN is still quite a rare complication which makes it difficult to evaluate study a large sample sizes.

A challenge in this study was to define an appropriate reference standard. CN is a clinical diagnosis that can be supported by imaging [10]. MRI could have been a suitable modality to serve as a reference standard [11, 12]. Due to the retrospective study design, MRI was not available. The long waiting times in clinical practice are detrimental for a rapid diagnosis, therefore clinicians did not opt for MRI. The final clinical diagnoses and a follow-up of three months was used as a reference standard. The accuracy of DECT in this patient population must be further investigated in a prospective setting.

VNCa images calculated from DECT are of potential value to assess the presence of BME in the midfoot of people with suspected CN.

## Data Availability

The data that support the findings of this study are available from the corresponding author upon reasonable request.

## References

[1] Fabrin J, Larsen K, Holstein PE. Long-term follow-up in diabetic Charcot feet with spontaneous onset. Diabetes Care. 2000;23:796–800. 10.2337/diacare.23.6.796. 10840999 10.2337/diacare.23.6.796

[2] Jeffcoate WJ. Charcot foot syndrome. Diabet Med. 2015;32:760–70. 10.1111/dme.12754. 25818542 10.1111/dme.12754

[3] Kaynak G, Birsel O, Güven MF, Oğüt T. An overview of the Charcot foot pathophysiology. Diabet Foot Ankle. 2013;4:21117. 10.3402/dfa.v4i0.21117. 10.3402/dfa.v4i0.21117 PMC3733015 23919113

[4] Jeffcoate W, Game F. The Charcot Foot Reflects a Response to Injury That Is Critically Distorted by Preexisting Nerve Damage: An Imperfect Storm. Diabetes Care. 2022;45:1691–7. 10.2337/dc21-2508. 35796768 10.2337/dc21-2508

[5] Cowley MS, Boyko EJ, Shofer JB, Ahroni JH, Ledoux WR. Foot ulcer risk and location in relation to prospective clinical assessment of foot shape and mobility among persons with diabetes. Diabetes Res Clin Pract. 2008;82:226–32. 10.1016/j.diabres.2008.07.025. 18829126 10.1016/j.diabres.2008.07.025

[6] Chantelau E. The perils of procrastination: effects of early vs. delayed detection and treatment of incipient Charcot fracture. Diabet Med. 2005;22:1707–12. 10.1111/j.1464-5491.2005.01677.x. 16401316 10.1111/j.1464-5491.2005.01677.x

[7] Wukich DK, Sung W, Wipf SA, Armstrong DG. The consequences of complacency: managing the effects of unrecognized Charcot feet. Diabet Med. 2011;28:195–8. 10.1111/j.1464-5491.2010.03141.x. 21219429 10.1111/j.1464-5491.2010.03141.x

[8] Keukenkamp R, Busch-Westbroek TE, Barn R, Woodburn J, Bus SA. Foot ulcer recurrence, plantar pressure and footwear adherence in people with diabetes and Charcot midfoot deformity: A cohort analysis. Diabet Med. 2021;4:e14438. 10.1111/dme.14438. 10.1111/dme.14438 PMC8048542 33084095

[9] Kavitha KV, Patil VS, Sanjeevi CB, Unnikrishnan AG. New Concepts in the Management of Charcot Neuroarthropathy in Diabetes. Adv Exp Med Biol. 2021;1307:391–415. 10.1007/5584_2020_498. 32124412 10.1007/5584_2020_498

[10] D.K. Wukich, N.C. Schaper, C. Gooday, A. Bal, R. Bem, A. Chhabra, M. Hastings, C. Holmes, N.L. Petrova, M.G. Santini Araujo, E. Senneville, K.M. Raspovic, 2023. Guidelines on the diagnosis and treatment of active Charcot neuro-osteoarthropathy in persons with diabetes mellitus (IWGDF 2023). Diabetes Metab. Res. Rev. e3646. 10.1002/dmrr.3646. 10.1002/dmrr.3646 37218537

[11] Schoots IG, Slim FJ, Busch-Westbroek TE, Maas M. Neuro-osteoarthropathy of the foot-radiologist: friend or foe? Semin Musculoskelet Radiol. 2010;14:365–76. 10.1055/s-0030-1254525. 20539961 10.1055/s-0030-1254525

[12] Short DJ, Zgonis T. Medical Imaging in Differentiating the Diabetic Charcot Foot from Osteomyelitis. Clin Podiatr Med Surg. 2017;34:9–14. 10.1016/j.cpm.2016.07.002. 27865318 10.1016/j.cpm.2016.07.002

[13] Wukich DK, Raspovic K, Liu GT, van Pelt MD, Lalli T, Chhabra A, Nakonezny P, La Fontaine J, Lavery L, Kim PJ. Are the Sanders-Frykberg and Brodsky-Trepman Classifications Reliable in Diabetic Charcot Neuroarthropathy? J Foot Ankle Surg. 2021;60:432–5. 10.1053/j.jfas.2020.03.003. 33549422 10.1053/j.jfas.2020.03.003

[14] Rosskopf AB, Loupatatzis C, Pfirrmann CWA, Böni T, Berli MC. The Charcot foot: a pictorial review. Insights Imaging. 2019;10:77. 10.1186/s13244-019-0768-9. 31385060 10.1186/s13244-019-0768-9 PMC6682845

[15] Sanverdi SE, Ergen BF, Oznur A. Current challenges in imaging of the diabetic foot. Diabet Foot Ankle. 2012;3:18754. 10.3402/dfa.v3i0.18754. 10.3402/dfa.v3i0.18754 PMC3464078 23050068

[16] Chen M, Herregods N, Jaremko JL, Carron P, Elewaut D, van den Bosch F, Jans L. Bone marrow edema in sacroiliitis: detection with dual-energy CT. Eur Radiol. 2020;30:3393–400. 10.1007/s00330-020-06670-7. 32055947 10.1007/s00330-020-06670-7

[17] Grunz JP, Sailer L, Lang P, Schüle S, Kunz AS, Beer M, Hackenbroch C. Dual-energy CT in sacral fragility fractures: defining a cut-off Hounsfield unit value for the presence of traumatic bone marrow edema in patients with osteoporosis. BMC Musculoskelet Disord. 2022;23:724. 10.1186/s12891-022-05690-2. 35906573 10.1186/s12891-022-05690-2 PMC9336065

[18] Guggenberger R. Dual-Energy CT in the Detection of Bone Marrow Edema in the Sacroiliac Joints: Is There a Case for Axial Spondyloarthritis? Radiology. 2019;290:165–6. 10.1148/radiol.2018182224. 30351250 10.1148/radiol.2018182224

[19] Foti G, Catania M, Caia S, Romano L, Beltramello A, Zorzi C, Carbognin G. Identification of bone marrow edema of the ankle: diagnostic accuracy of dual-energy CT in comparison with MRI. Radiol Med. 2019;124:1028–36. 10.1007/s11547-019-01062-4. 31273545 10.1007/s11547-019-01062-4

[20] Wang CK, Tsai JM, Chuang MT, Wang MT, Huang KY, Lin RM. Bone marrow edema in vertebral compression fractures: detection with dual-energy CT. Radiology. 2013;269:525–33. 10.1148/radiology.13122577. 23801776 10.1148/radiology.13122577

[21] Mens MA, de Geus A, Wellenberg RHH, Streekstra GJ, Weil NL, Bus SA, Busch-Westbroek TE, Nieuwdorp M, Maas M. Preliminary evaluation of dual-energy CT to quantitatively assess bone marrow edema in patients with diabetic foot ulcers and suspected osteomyelitis. Eur Radiol. 2023. 10.1007/s00330-023-09479-2. 36820925 10.1007/s00330-023-09479-2 PMC10326105

[22] Marmolejo VS, Arnold JF, Ponticello M, Anderson CA. Charcot Foot: Clinical Clues, Diagnostic Strategies, and Treatment Principles. Am Fam Physician. 2018;97:594–9. 29763252

[23] Brodsky JW, Rouse AM. Exostectomy for symptomatic bony promineces in diabetic charcot feet. Clin Orthop Res. 1993;(296):21–6. 8222428

[24] Trepman E, Nihal A, Pinzur MS. Current topics review: Charcot neuroarthropathy of the foot and ankle. Foot Ankle Int. 2005;26:46–63. 10.1177/107110070502600109. 15680119 10.1177/107110070502600109

[25] Robinson AH, Pasapula C, Brodsky JW. Surgical aspects of the diabetic foot. J Bone Joint Surg Br. 2009;91:1–7. 10.1302/0301-620X.91B1.21196. 19091997 10.1302/0301-620X.91B1.21196

[26] Koo TK, Li MY. A Guideline of Selecting and Reporting Intraclass Correlation Coefficients for Reliability Research. J Chiropr Med. 2016;15:155–63. 10.1016/j.jcm.2016.02.012. 27330520 10.1016/j.jcm.2016.02.012 PMC4913118

[27] Landis JR, Koch GG. The measurement of observer agreement for categorical data. Biometrics. 1977;33:159–74. 843571

[28] Chantelau E, Richter A, Schmidt-Grigoriadis P, Scherbaum WA. The diabetic charcot foot: MRI discloses bone stress injury as trigger mechanism of neuroarthropathy. Exp Clin Endocrinol Diabetes. 2006;114:118–23. 10.1055/s-2006-924026. 16636977 10.1055/s-2006-924026

[29] Foti G, Beltramello A, Catania M, Rigotti S, Serra G, Carbognin G. Diagnostic accuracy of dual-energy CT and virtual non-calcium techniques to evaluate bone marrow edema in vertebral compression fractures. Radiol Med. 2019;124:487–94. 10.1007/s11547-019-00998-x. 30712165 10.1007/s11547-019-00998-x

[30] Guggenberger R, Gnannt R, Hodler J, Krauss B, Wanner GA, Csuka E, Payne B, Frauenfelder T, Andreisek G, Alkadhi H. Diagnostic performance of dual-energy CT for the detection of traumatic bone marrow lesions in the ankle: comparison with MR imaging. Radiology. 2012;264:164–73. 10.1148/radiol.12112217. 22570505 10.1148/radiol.12112217

[31] Chen Z, Chen Y, Zhang H, Jia X, Zheng X, T. Zuo T,. Diagnostic accuracy of dual-energy computed tomography (DECT) to detect non-traumatic bone marrow edema: A systematic review and meta-analysis. Eur J Radiol. 2022;153:110359. 10.1016/j.ejrad.2022.110359. 35609447 10.1016/j.ejrad.2022.110359

